# Freitagnachts unterwegs im ÖPNV: Muster und Motive anhand des Fallbeispiels Karlsruhe

**DOI:** 10.1007/s00548-022-00774-6

**Published:** 2022-04-12

**Authors:** Jonas Kapitza

**Affiliations:** Institut für Geographie und Geoökologie, Karlsruher Institut für Technologie, Kaiserstraße 12, Gebäude 10.50, 76131 Karlsruhe, Deutschland

**Keywords:** Nächtliche Mobilität, Verkehrsmittelwahl, Freizeitmobilität, Nächtliche Ökonomien, Rhythmusanalyse, Night-time mobility, Mode choice, Leisure mobility, Night-time economies, Rhythmanalysis

## Abstract

Urbane Zentren spielen eine zentrale Rolle für nächtliche Freizeitaktivitäten in der Stadt. In ihnen bündeln sich die Angebote der nächtlichen Freizeitökonomien, wie Bars, Restaurants oder Clubs, die oft auf einzelne Hotspots konzentriert sind. Um zu diesen Hotspots zu gelangen, müssen Wege zurückgelegt und Verkehrsmittel genutzt werden. Dabei unterscheidet sich die räumliche Mobilität in der Nacht, insbesondere im Bereich des öffentlichen Personennahverkehrs (ÖPNV), in vielerlei Hinsicht von derjenigen am Tag. Vor diesem Hintergrund analysiert der Beitrag am Beispiel von Karlsruhe die nächtlichen Mobilitätsprozesse, die zu Freizeitzwecken im ÖPNV stattfinden. Die Basis der Analyse bildet ein Datensatz, der im Rahmen eines studentischen Workshops während einer nächtlichen Befragung in den Bahnen der Karlsruher Verkehrsbetriebe entstanden ist. Die Ergebnisse zeigen unter anderem, dass Haltestellen, die sich in unmittelbarer Nähe der nächtlichen Freizeitökonomien befinden, in der Nacht überdurchschnittlich oft von Fahrgästen des öffentlichen Personennahverkehrs frequentiert werden. Trotz einiger Kritikpunkte wird das nächtliche ÖPNV-Angebot dabei überwiegend positiv wahrgenommen. Gleichwohl zeigt der Artikel grundsätzliche Verbesserungspotenziale auf und stellt geeignete Handlungsspielräume und Optimierungsmaßnahmen vor. Abschließend werden Empfehlungen für weitere Forschungsansätze zum Thema der nächtlichen Mobilität gegeben.

## Abstract (en)

Urban centers play a central role in night-time leisure activities in the city. They combine the offerings of the night-time economies, such as bars, restaurants or clubs, which are often concentrated in separate hotspots. To get to these hotspots, routes have to be travelled and means of transport have to be used. In this context, spatial mobility at night, especially in the field of local public transport, differs in many respects from that during the day. Against this background, the article analyzes the nocturnal mobility processes that take place for leisure purposes on public transport, using Karlsruhe as an example. The basis of the analysis is a data set that was created as part of a student workshop during a nightly survey of the trains of the Karlsruhe public transport system. The results show, among other things, that stops located in the immediate vicinity of night-time economies are used more frequently than average by public transport passengers during the night. Despite some points of criticism, the public transport service at night is largely perceived positively. Nevertheless, the article identifies fundamental potential for improvement and presents suitable scope for action and optimization measures. Finally, recommendations for further research approaches to the topic of night-time mobility are given.

## Nächtliche Mobilität in der Stadt

Die Nacht als Raum ist längst zu einem wichtigen Bestandteil des städtischen Lebens geworden. Immer mehr Praktiken des Alltags verlagern sich in die Zeit nach Einbruch der Dunkelheit und sorgen somit dafür, dass die Nacht zunehmend zu einem sozial, aber auch ökonomisch relevanten (Zeit‑)Raum avanciert. Eine räumlich differenzierte Ansiedlung nächtlicher Ökonomien soll Personen nicht nur tagsüber, sondern gezielt auch nachts in die urbanen Zentren locken und so zu einer Belebung und Revitalisierung der Innenstädte beitragen (Thomas und Bromley 2000). Insbesondere die Wochenendnächte gelten dabei als Zeitperioden von gesteigertem Interesse (Kapitza 2020). So stellt u. a. Schäfer (2020, S. 101) fest, dass die „zeitlichen Nutzungsschwerpunkte [der Innenstadtgastronomie] an Samstagen und Freitagen in den Abendstunden“ liegen. Hollands (1995, S. 8) konstatiert, dass „[e]ven a casual observer could not fail to notice a ritual descent of young adults onto the city centre streets at least every Friday and Saturday night“.

Ein wichtiger Aspekt, der dabei oft übersehen wird, ist die räumliche Mobilität. Personen, die nachts unterwegs sind, müssen Wege zurücklegen und Verkehrsmittel nutzen. Dabei unterscheidet sich die räumliche Mobilität in der Nacht, insbesondere im Bereich des öffentlichen Personennahverkehrs (ÖPNV), in vielerlei Hinsicht von derjenigen am Tag. Beispielsweise wird das Angebot des ÖPNVs in der Nacht im Gegensatz zum Tag oft als defizitär eingestuft (Schäfer 2020; Gwiazdzinski 2007). Darüber hinaus gilt der ÖPNV im Vergleich zu anderen Verkehrsmitteln als weniger sicher (Yavuz und Welch 2010; Currie et al. 2021).

Obwohl die Nacht als Untersuchungs(zeit)raum in letzter Zeit verstärkt das wissenschaftliche Interesse auf sich gezogen hat, ist die räumliche Mobilität in der Nacht noch weitgehend unerforscht. Der vorliegende Beitrag versucht, dieses Defizit zu verringern und konzentriert sich dabei auf die Alltagsmobilität zum Zweck der Freizeitgestaltung. Am Beispiel von Karlsruhe wird u. a. untersucht, wie sich die nächtliche Mobilität im Raum darstellt, aus welchen Gründen der ÖPNV als Verkehrsmittel gewählt wird und wie er hinsichtlich seiner Nutzung in der Nacht bewertet wird. Die Stadtnacht selbst ist dabei als ein Raum-Zeit-Modell zu verstehen, das sowohl in räumlichen als auch zeitlichen Dimensionen gedacht und interpretiert werden will (Schlör 1994). Vor diesem Hintergrund greift die folgende Studie auf das theoretische Konzept der Rhythmusanalyse zurück, durch das raumzeitliche Strukturen in der Nacht mit dem Alltagsleben kontextualisiert werden können.

## Die städtische Nacht im Kontext von Raum, Zeit und Rhythmus

Der französische Philosoph und Soziologe Henri Lefebvre gilt als Begründer der Rhythmusanalyse. Er vertritt die These, dass Wechselwirkungen zwischen Raum und Zeit, durch das Konzept des Rhythmus beschrieben werden können (Lefebvre 2004). Nach seiner Konzeption ist es wichtig, sowohl Raum als auch Zeit als soziale Konstrukte zu verstehen, die in ständiger Interaktion zueinanderstehen und basierend auf der Art und Weise, wie Menschen sich zueinander und zu ihrer Umwelt verhalten, gesellschaftlich produziert und konsumiert werden können (Alhadeff-Jones 2019). Vor diesem Hintergrund kann das urbane Zentrum als ein Raum verstanden werden, der im wiederkehrenden Rhythmus von Tag und Nacht einem Puls unterschiedlicher Nutzungsaspekte, Handlungserwartungen und Bedeutungszuweisungen unterliegt, die sich immer auch aus den Beziehungen zu den Menschen ergeben, die sich in diesem Raum aufhalten.

In einer raumzeitlichen Betrachtung ist zu erkennen, dass die Innenstädte am Tag das Zentrum des geschäftlichen Treibens bilden. Mit dem Einbruch der Nacht nimmt diese Betriebsamkeit ab, und die urbanen Aktivitäten verlagern sich von der Fläche auf einzelne, oft dezentral strukturierte Orte (Gwiazdzinski 2015; Weber und Henckel 2019). Diese Orte formen monofunktionale Hotspots vergnügungsgebundener Aktivitäten, die sich durch eine hohe Dichte an Einrichtungen der sogenannten *Night Time Economy (NTE)*, wie Bars, Restaurants oder Clubs auszeichnen und die Hauptanziehungspunkte des urbanen Nachtlebens bilden. Im Übergang zwischen Tag und Nacht ändert sich die Raumnutzung und somit das Verhältnis zwischen den im Raum agierenden Akteuren und ihrer substanziellen Umwelt. Gleichzeitig mit den Nutzungsaspekten verändern sich auch die Bedeutungszuschreibungen und Handlungserwartungen, die an den innerstädtischen Raum gestellt werden.

**Abb. 1:**
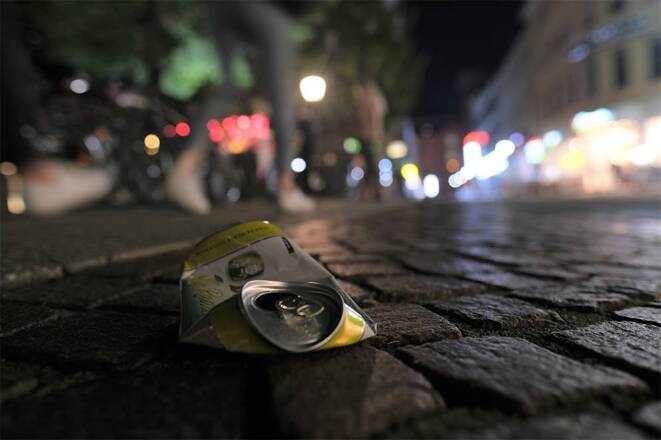
Spuren der nächtlichen Partygäste in der Karlsruhe Fußgängerzone. (Foto: Barkawitz 2021)

Nicht selten führt die freizügige Atmosphäre der Stadtnacht bei der feiernden Partygesellschaft zu einer Loslösung von denen am Tage noch bestehenden Konventionen. Van Liempt et al. (2015, S. 408) stellen fest, dass „Nightlife areas with bars and clubs are often emotionally charged spaces offering many chances for the transgression of social norms that are take for granted during the day.“ Der Konsum von Alkohol und Drogen kann diesen Prozess verstärken und zu einem weiteren Kontroll- und Normverlust aufseiten der Konsumenten führen (Nelson et al. 2001). Ein soziales Verantwortungsbewusstsein oder eine wertschätzende Sinnzuschreibung an die bestehende Umwelt bleiben dann oft aus. Die Folgen sind ein achtloser Umgang im und mit dem öffentlichen Raum, der sich u. a. in einem erhöhten Müllaufkommen, vorsätzlichen Verschmutzungen oder Vandalismus äußert (vgl. Abb. 1; Weber und Henckel 2019). Nicht selten werden diese Eigenschaften auch auf die öffentlichen Verkehrsmittel projiziert und setzen sich in diesen fort (Currie et al. 2021).

Gelegentlich geht der Prozess des Normenverlusts mit einem aggressiven Verhalten gegenüber Mitmenschen oder anderen Fahrgästen einher, was die Frage der Sicherheit zu einem zentralen Thema der Nachtforschung macht (Hadfield 2009; van Liempt und van Aalst 2011). Die Forschungen zeigen, dass Gewalttaten an bestimmten Orten und zu bestimmten Zeiten Auswirkungen auf das Sicherheitsempfinden in und um diese (Zeit‑)Räume haben. Wenn Gewalttaten beispielsweise vermehrt an Haltestellen stattfinden, manifestieren sich Gedankenbilder, die diese Räume von sich aus unsicher erscheinen lassen (Nelson et al. 2001). Das Vorhandensein von visuellen Symbolen der Kriminalität, wie z. B. Verschmutzungen oder Zeichen von Vandalismus, intensivieren diese mentalen Konstruktionen zusätzlich (Currie et al. 2021).

Im vorliegenden Beitrag gilt es daher, folgende Annahmen zu prüfen:

Durch die Verschiebung der nächtlichen Aktivitäten auf wenige Ballungszentren, kann davon ausgegangen werden, dass die Haltestellen, die sich in fußläufiger Entfernung zu diesen Ausgehvierteln befinden, in den frühen Nachtstunden eine höhere Fahrgastfrequenz aufweisen als die umliegenden Haltestellen. Personen, die zu ihrer nächtlichen Freizeitgestaltung in die Städte einpendeln, steigen vermehrt an diesen Haltestellen aus und Personen, die auf dem Rückweg sind, steigen vermehrt an diesen ein. Des Weiteren besteht die Annahme, dass gerade alkoholisierte Personen auf die nächtlichen ÖPNV-Angebote zurückgreifen, da sie aufgrund ihrer Fahruntauglichkeit viele alternative Verkehrsmittel nicht nutzen können oder dürfen. Die öffentlichen Verkehrsmittel werden dabei oft nur als temporärer Aufenthaltsort wahrgenommen, die zum reinen Zweck der Fortbewegung dienen. Besonders an Tagen, an denen die Partygesellschaft im innerstädtischen Raum aktiv ist, ist daher in Anlehnung an die aufgeführte Theorie von Weber und Henckel (2019) mit einer erhöhten Verschmutzung in den Bussen und Bahnen der öffentlichen Verkehrsbetriebe zu rechnen. Die Mobilitätsforschung hat gezeigt, dass das Fahren im ÖPNV mit zu den am stärksten angstauslösenden Aktivitäten im öffentlichen Raum zählt (Yavuz und Welch 2010). Das mutmaßlich erhöhte Verschmutzungsaufkommen und der vermeintlich gestiegene Vandalismus, ausgelöst durch die freizügige Atmosphäre in der Nacht, können diese Wahrnehmung verstärken. Daraus resultiert die Annahme, dass die öffentlichen Verkehrsmittel im Untersuchungszeitraum ebenfalls als eher unsicher wahrgenommen und auch so bewertet werden.

## Methodisches Vorgehen

Um die formulierten Annahmen zu überprüfen, wird ein Datensatz analysiert, der im Rahmen eines studentischen Workshops in der Nacht vom Freitag, 26. Juni, auf Samstag, 27. Juni 2020, entstanden ist. Zwischen 22:00 Uhr abends und 01:00 Uhr morgens waren 22 Studierende auf 6 verschiedenen Routen innerhalb des Karlsruher Stadtbahnnetzes unterwegs. Bei der Routenauswahl wurde darauf geachtet, das innerstädtische Untersuchungsgebiet so gut wie möglich abzudecken. Gleichzeitig sollten längere Wartezeiten an den Haltestellen und Doppelbesetzungen der Stadtbahnen vermieden werden. Während der Erhebung wurden die Fahrgäste nach dem Zufallsprinzip ausgewählt und unter der Verwendung eines halbstandardisierten Fragebogens in Form eines Pen-and-Paper-Interviews befragt. Der Fragebogen enthielt 15 offene und geschlossene Fragen zur Strecken- und Verkehrsmittelwahl, zur Wahrnehmung der Fahrt und zu soziodemografischen Merkmalen. Ein Problem bei der Befragung war, dass die durchschnittliche Befragungsdauer von knapp 5 min die Fahrtzeit einiger Fahrgäste überstieg. Passagiere, die die Bahnen nur für wenige Haltestellen nutzten, konnten daher nicht in die Befragung einbezogen werden. Gleichwohl wurden Daten von *n* = 176 Personen im Alter von 16–72 Jahren erhoben. Im Anschluss an den Workshop wurden die Daten mittels SPSS nachcodiert und statistisch ausgewertet.

## Nächtliche Mobilität im Karlsruher ÖPNV

**Abb. 2:**
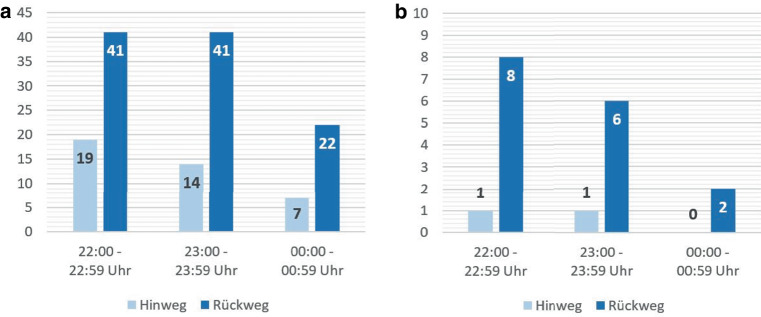
Anzahl an Hin- und Rückwegen gruppiert im Stundentakt nach Wegezwecken (**a**) Freizeit und (**b**) Arbeit. (*n* = 162)

Die Ergebnisse der Datenanalyse zeigen, dass während der frühen Freitagnacht 86 % der Passagiere aus Gründen der Freizeit unterwegs waren. Je weiter die Nacht fortschritt, desto mehr ließ das Personenaufkommen nach. Dabei befanden sich jederzeit mehr Personen auf dem Rück- als auf dem Hinweg ihrer Aktivität (vgl. Abb. 2). Eine Ursache hierfür könnten die erlassenen Eindämmungsmaßnahmen der COVID-19-Pandemie sein. In der Zeit zwischen der sogenannten ersten und zweiten „Coronawelle“, während der diese Befragung stattfand, durften Gastronomiebetriebe zwar öffnen, innerräumliche Partylocations, wie Clubs, Diskotheken o. Ä. mussten aber geschlossen bleiben. Die Folge war ein stark reduziertes Angebot der NTE, das sich mit hoher Wahrscheinlichkeit auch in der Nutzungsintensität des ÖPNVs und der Anzahl der Wege ins Stadtzentrum, insbesondere zu späterer Stunde, niederschlug. Eine von den Karlsruher Verkehrsbetrieben intern ausgeführte Datenauswertung untermauert diese Erkenntnis und zeigt, dass zu dieser Zeit im Durchschnitt zwischen 22:00 Uhr und 02:00 Uhr an den innerstädtischen Haltestellen 930 Einsteiger 486 Aussteigern gegenüberstehen (VBK 2020^1^).

**Abb. 3:**
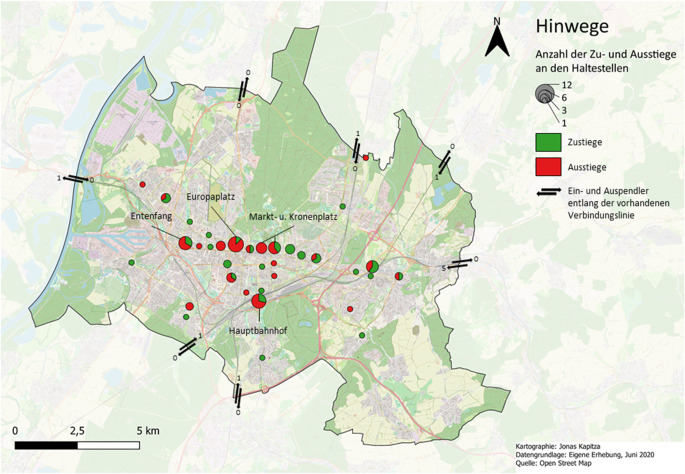
Anzahl der Zustiege (*grün*) und Ausstiege (*rot*) an den kartierten Haltestellen im Stadtkreis Karlsruhe von Personen auf dem Hinweg. (*n* = 44)

Dennoch zeigt die vorliegende Datenauswertung, dass die Standorte in der Karlsruher Innenstadt, die besonders stark von der NTE geprägt sind, im Vergleich zu den umliegenden Orten in der Nacht überdurchschnittlich oft frequentiert wurden. Insbesondere die Orte rund um die Haltestellen Entenfang, Europaplatz, Markt- und Kronenplatz sowie Hauptbahnhof wiesen eine hohe Anzahl an Ausstiegen bei den Hinwegen, bei einer gleichzeitig hohen Anzahl an Zustiegen bei den Rückwegen auf (vgl. Abb. 3 und 4). Alle 4 Standorte zeichnen sich durch eine hohe Dichte an nächtlichen Ökonomien wie Bars und Restaurants aus (vgl. Abb. 5).

**Abb. 4:**
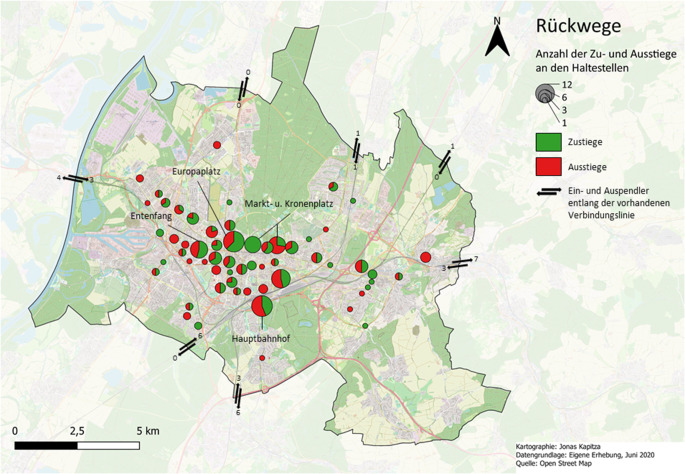
Anzahl der Zustiege (*grün*) und Ausstiege (*rot*) an den kartierten Haltestellen im Stadtkreis Karlsruhe von Personen auf dem Rückweg. (*n* = 127)

**Abb. 5:**
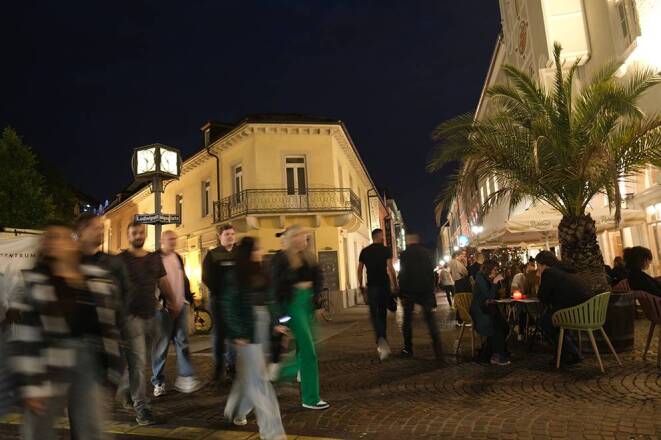
Nächtliches Treiben am Ludwigsplatz, nahe der Haltestelle Europaplatz. (Foto: Barkawitz 2021)

Tab. 1 gibt einen Überblick über die Relationen zwischen den Wegezwecken und den Gründen für die Wahl des ÖPNVs als Verkehrsmittel. Dabei zeigen sich signifikante Zusammenhänge zwischen den Wegezwecken und den Variablen „Alternativlosigkeit“ und „Fahruntauglichkeit“. Das Cramér’sche Assoziationsmaß V bezieht sich auf die Stärke der Ausprägungen dieser Zusammenhänge. Werte von 0,25 und 0,16 weisen unter der Prämisse der Signifikanz auf schwach bis mäßig ausgeprägte Zusammenhänge hin (Duller 2019). Hinsichtlich des ersten Zusammenhangs ist festzustellen, dass mehr als die Hälfte (59 %) der Personen, die sich auf dem Arbeitsweg befinden, angeben, dass sie mangels Alternativen keine andere Möglichkeit sehen, als den ÖPNV für ihren Weg zu nutzen. Dabei nennen sie als Ursache, dass sie entweder kein Auto oder Führerschein besitzen oder aber, dass ihnen anderweitig der Zugang zu einem alternativen Verkehrsmittel verwehrt ist. 71 % dieser Personen geben an, den ÖPNV „immer“ für ihre Arbeitswege zu nutzen. Anders verhält es sich bei den Personen, die aus Gründen der Freizeit unterwegs sind. Hier geben lediglich 35 % an, den ÖPNV „immer“ für diesen Wegezweck zu nutzen. Ungefähr genauso viele Personen (37 %) nutzen den ÖPNV „gelegentlich“, „selten“ oder „fast nie“. Daraus lässt sich schließen, dass innerhalb der Freizeitmobilität mit einem breiteren Spektrum an Verkehrsmitteln zu rechnen ist, zwischen denen gleichzeitig häufiger variiert wird.

**Tab. 1:** Gründe der Verkehrsmittelnutzung nach Wegezweck (Mehrfachantworten möglich)

Gründe der Verkehrsmittelwahl (Angaben in %)	Fahruntauglichkeit	Bequemlichkeit	Alternativlosigkeit	Ticket	Schnelligkeit	Wetter	Sonst
*Wegezweck*							
Arbeit	0,0	23,5	58,8	17,6	5,9	0,0	5,9
Freizeit	20,1	33,3	22,9	18,8	9,7	8,3	19,4
**Gesamt**	**18,0**	**32,3**	**26,7**	**18,6**	**9,3**	**7,5**	**18,0**
Cramér’s V	0,16**	0,06	0,25***	0,01	0,04	0,10	0,11

*n* = 161, $$X ^{2}=18,6^{***}$$, eigene Berechnung, *p < 0,1 **p < 0,05 ***p < 0,01

Hinsichtlich der Wahlmotive geben ein Drittel (32 %) aller Befragten an, dass der ÖPNV im Vergleich zu anderen Verkehrsmitteln am einfachsten und/oder bequemsten zu nutzen sei. Darüber hinaus zeigt das Cramér’sche Assoziationsmaß, dass die „Fahruntauglichkeit“ ein signifikant ausgeprägtes Kriterium dafür ist, dass viele Personen, die nachts zu Freizeitzwecken unterwegs sind, die Busse und Bahnen im Karlsruher Stadtgebiet nutzen. Ganze 20 % äußern, dass sie aufgrund ihres Alkoholkonsums nicht mehr dazu fähig sind, andere Verkehrsmittel zu benutzen und daher auf die Straßenbahn zurückgreifen. Damit bestätigt sich die Annahme, dass gerade alkoholisierte Personen vermehrt das nächtliche ÖPNV-Angebot am Wochenende wahrnehmen.

**Abb. 6:**
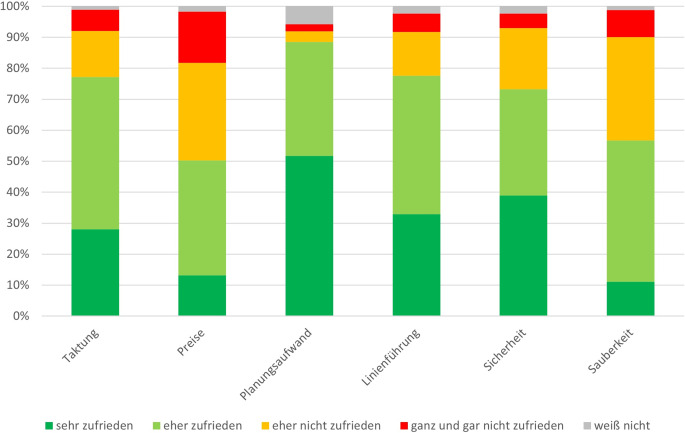
Zufriedenheit der befragten Personen mit den aufgeführten Eigenschaften des ÖPNVs. (*n* = 173)

Auch die These, dass an Tagen, an denen die feiernde Partygesellschaft im innerstädtischen Raum aktiv ist, mit einer erhöhten Verschmutzung im ÖPNV zu rechnen ist, kann mit den Ergebnissen der Analyse untermauert werden. Abb. 6 zeigt einen Überblick über die Bewertung bestimmter Aspekte seitens der befragten Fahrgäste. Hierbei zeigt sich, dass 42 % mit der Sauberkeit „eher nicht“ oder „ganz und gar“ nicht zufrieden sind, was nach der Zufriedenheit mit den Preisen, die in etwa von der Hälfte aller Befragten kritisiert werden, der zweitgrößte Kritikpunkt ist. Ähnlich verhält es sich mit dem Aspekt der Sicherheit. Hier zeigt sich, dass ein Viertel der befragen Personen die Bahnen als eher unsicher bzw. ganz und gar nicht sicher wahrnehmen. Als Hauptgründe werden ein in der Nacht oft unangenehmes Klientel und das Problem der Einengung bzw. der fehlenden Fluchtmöglichkeit angegeben. Die Aussagen bestätigen damit die bereits vorhandenen Erkenntnisse der Literatur (vgl. u. a. Yavuz und Welch 2010). Allerdings muss an dieser Stelle festgehalten werden, dass sich die Beurteilungen der Aspekte im insgesamt positiven Bereich bewegen. Besonders hoch ist die Zufriedenheit mit der Linienführung sowie der Taktung der Bahnen, was sich auch in der Zufriedenheit mit dem Planungsaufwand widerspiegelt. Fast 90 % bewerten den Aufwand, den sie bei der Planung ihrer Wege mit dem ÖPNV haben, positiv.

## Fazit

Das Beispiel der Karlsruher Innenstadt zeigt, wie sich räumliche Nutzungsaspekte und Bedeutungszuweisungen in einem zeitlichen Rhythmus verändern können. Während tagsüber im Stadtzentrum meist flächendeckend eine rege Betriebsamkeit herrscht, verlagert sich das lebendige Umfeld nachts auf wenige, räumlich dezentral organisierte Hotspots. Die Studienergebnisse zeigen, dass genau diese Orte die primären Ziele der Personen sind, die freitagnachts zum Zweck der Freizeit den ÖPNV benutzen. Gleichzeitig bilden diese Orte auch den zentralen Ausgangspunkt für die Personen, die sich auf dem Rückweg ihrer Freizeitaktivität befinden. Das Angebot des Karlsruher ÖPNVs wird dabei überwiegend positiv wahrgenommen. Optimierungsbedarf gibt es lediglich bei den Ticketpreisen, der Sauberkeit und der Sicherheitswahrnehmung. Generell darf davon ausgegangen werden, dass eine Reduzierung der Verschmutzungen bzw. der Anzeichen von Vandalismus dazu beitragen würde, sowohl das Sicherheitsgefühl als auch die Gesamtzufriedenheit der Fahrgäste weiter zu erhöhen. Hier haben die Verkehrsbetriebe noch Handlungsspielraum, um die öffentliche Wahrnehmung des ÖPNVs als Fortbewegungsmittel in der Nacht weiter zu steigern.

Hinsichtlich der angewandten Methode lässt sich konstatieren, dass eine Ausweitung auf andere Beobachtungszeiträume die Qualität und Aussagekraft der Daten noch weiter verbessern würde. Hierdurch wäre ein Vergleich möglich, der die Merkmale der nächtlichen Mobilität von denen anderer Zeiträume abgrenzt. Auch eine Gegenüberstellung mit Daten von ergänzenden Erhebungen aus anderen Städten erscheint sinnvoll und lohnenswert. An diesen Punkten gilt es in Zukunft anzusetzen, um das Forschungsfeld der nächtlichen Mobilität weiter zu stärken.

## Footnotes

1 VBK (2019) [interne, unveröffentlichte Datenanalyse zu Ein- und Aussteigern an den Haltestellen Kronenplatz (Kaiserstr.), Marktplatz, Herrenstraße und Europaplatz (Kaiserstr.) zw. 11.05.2020 bis 04.10.2020 von 22:00–02:00 Uhr, Erfasste Linien: 1, 4, S1, S11, S2, S5, S51] Verkehrsbetriebe Karlsruhe.
